# The correction factor of Monterey No. 0/30 sample with fines content liquefaction resistance between cyclic triaxial and cyclic hollow cylinder tests

**DOI:** 10.1038/s41598-022-20002-3

**Published:** 2022-09-23

**Authors:** Jungang Liu, Geng Chen

**Affiliations:** 1 Department of Civil Engineering, College of Civil Engineering and Architecture at the Hubei Polytechnic University, No. 16 North of Guilin Road, K8-206, Huangshi, 435003 Hubei China; 2 Center for Geotechnical Engineering Science, University of Colorado Denver, Denver, CO USA; 3 Department of Civil Engineering, University of Colorado Denver, 923 South Fairplay Street, Aurora, CO 80012 USA; 4 Center for Geotechnical Engineering Science, University of Colorado Denver, Aurora, CO USA

**Keywords:** Civil engineering, Natural hazards

## Abstract

Soil liquefaction is one of the most detrimental forms of earthquake-induced ground failure that can result in catastrophic damage to engineering structures. For the seismic safety evaluation of foundations and high-rise structures, it is the most critical way to assess liquefaction resistance. In this paper, an array number of isotropically consolidated undrained cyclic triaxial tests (CTT) and cyclic hollow cylinder tests (CHCT) have been performed to evaluate soil liquefaction resistance. Thirty-seven isotopically consolidated undrained cyclic triaxial tests and thirty-seven cyclic hollow cylinder tests were run on the uniform medium Monterey No. 0/30 sand and it is with four different percentages of fine content. By using cyclic triaxial and cyclic hollow cylinder tests for evaluating liquefaction resistance, it helps us better understand the relationship between two types of tests on uniform clean Monterey No. 0/30 sand and soil sample with five different percentages of fines content. Four different relative densities of 30%, 45%, 50%, and 60%, two confining pressure of 103 kpa and 207 kpa, and five cyclic stress ratios (0.15, 0.2, 0.25, 0.3, and 0.4) have been used for a series of cyclic triaxial tests and cyclic hollow cylinder tests. At the same relative densities of 30% and 60%, the correction factor between CTT and CHCT evaluated ranged from 0.46 to 0.63 on the uniform clean Monterey No. 0/30 sand. Statistical analyses were performed to formulate functional relationships for predicting the correction factor of soil liquefaction resistance between CTT and CHCT tests.

## Introduction

Soil liquefaction is a ground failure or loss of strength that causes solid soil to behave temporarily as a viscous liquid. It is long enough for liquefaction to be the cause of many failures, deaths, and major financial losses. When shaken by earthquakes, saturated loose granular soil will tend to density. Since the soil is saturated, the densification requires that water be expelled out of the soil mass so that soil particles can become more densely packed. If the process of expelling water cannot occur immediately, the soil particles will tend to become waterborne and induce a rapid rise in pore water pressure. When the induced pore water pressure becomes high enough to counterbalance the total stress acting on soil particles, the granular soil will lose all its shear strength and is, in a broad sense, designated as "in a state of liquefaction failure."

The tremendous damage due to soil liquefaction was observed in many earthquakes. Especially after the Alaska earthquake of 1964, the Niigata earthquake of 1964, the Tangshan earthquake of 1976, geotechnical engineering paid much research attention to studying earthquake-induced soil liquefaction (e.g. Ishihara and Koga^1^ and Roscoe et al.^2^).

At the same time, the liquefaction mechanism and phenomena have been investigated. However, due to the complexity of liquefaction problems, the method of an accurate evaluation of liquefaction resistance is still needed.

Soil liquefaction is one of the most important and complex issues studied in geotechnical earthquake engineering. Many scholars investigated the phenomenon and significant results were achieved (e.g. Liu^3^, Chen et al.^4^, Karakan et al.^5^ and Yang and Sze^6^, among many others). For many years, the phenomenon of liquefaction was thought to be only limited to sands. However, natural sandy soils contain fines (passing sieve No. 200, particle size less than 0.074 mm) more or less.

Therefore, the effect of the fines content (FC) on the liquefaction behavior of sand-fines mixtures has attracted increased interest from scholars in the past few decades and many test data are available in the technical literature. The approach of a precise evaluation of liquefaction resistance is still required because of the complexity of liquefaction questions.

An ideal laboratory testing apparatus should closely simulate initial stress and loading conditions in the field. Cyclic triaxial test, cyclic hollow cylinder test, and cyclic simple shear (CSS) tests are traditionally used in experiments for liquefaction resistance evaluation. However, CTT can only apply jump rotation on principal stresses, CSS has a disadvantage from the nonuniform distributions of stress and strain. With appropriate dimensions, specimens in CHCT not only have much more uniform distributions of stress and strain but also can be more ideally simulated field conditions. Thus, CHCT is considered an ideal test for the evaluation of liquefaction resistance. Dai^7^ showed that the cylindrical soil samples in CHCT has uniform stress and strain distributions.

This paper included (1) performing cyclic triaxial tests and cyclic hollow cylinder tests to evaluate the liquefaction resistance of soils and it is containing fines. (2) comparison of the results of liquefaction resistance of Monterey No. 0/30 sand and it is containing fines. (3) evaluate the correlation between CTT and CHCT. (4) developing statistical models for predicting the correction factor between the two types of tests.

### Previous studies

Several early investigations were focused on studying the behavior and strength of soil liquefaction by using the cyclic triaxial test or cyclic hollow cylinder test; however, none of the studies presented the comparison of both test results and correction factors from two tests for evaluating soil liquefaction resistance. These early studies involving hollow cylinder tests were indicated by Hight et al.^8^, who tabulated them as follows: Suklje and Drnovesk^9^, Wijewickreme and Vaid^10^, Jamal^11^, Dusseault^12^ and Lade and Rodriguez^13^.

The application of cyclic triaxial test and cyclic hollow cylinder shear test on the behavior and strength of soil liquefaction had been researched by early studies. however, none of these earlier studies presented the comparison of both test results on the same soil samples. Especially, correction factors from two tests for evaluating soil liquefaction resistance are still needed to evaluate. In this paper, it included the comparison of both test results on soil samples and it is containing fines, the evaluation of the correction factor, and also the statistical regression models the for correction factor were proposed.

Sivathayalan et al.^14^ studied the effects of principal stress rotation on cyclic resistance in hollow cylinder torsional shear tests. Lade and Rodriguez^13^ showed that the results of hollow cylinder tests and true triaxial tests on dense cross-anisotropic fine Nevada sand are compared.

In this study, a series number of isotropically consolidated undrained cyclic triaxial tests and cyclic hollow cylinder tests have been performed on Monterey No. 0/30 sand. The results of hollow cylinder tests and cyclic triaxial tests on different relative densities of Monterey No. 0/30 sand and containing fines are compared. Both test results and the reduction factor between CTT and CHCT are discussed in the following.

### Test materials and sample preparation

Two different soil types were used to study liquefaction resistance in cyclic triaxial and cyclic hollow cylinder tests, where fines contents ranged from five percent to thirty-five percent, and plasticity indices were twenty percent. These soil samples included Monterey sand and Leyden Clay.

A Monterey sand is a classical research clean sand from Monterey, California. Laboratory testing was performed to evaluate the (1) minimum unit weights, (2) specific gravity, (3) friction angle, and (4) standard (Proctor) compaction response of Monterey sand. The testing was performed in general accordance with ASTM International (ASTM) designations D4254 (2006), D854 (2014), D3080-04 (2012) and and D698 (2012b). The results of the testing are presented in Table 1. Monterey sand is classified as SP via the Unified Soil Classification System.

**Table 1:** Minimum unit weights, specific gravity, friction angle, and standard proctor values for Monterey sand.

Monterey sand (MS)	
Maximum unit weight	105.8 pcf (Ib/ft3) (6.6 kg/m3)
Minimum unit weight	91.7 pcf (Ib/ft^3^) (5.7 kg/m^3^)
Specific gravity	2.65
Friction angle	37°

The Leyden Clay that was used for the test was supplied by the Pioneer Sand Company from a site near Golden, Colorado. Laboratory testing was performed to evaluate the (1) Atterberg limits, (2) specific gravity, and (3) standard (Proctor) compaction response of the Leyden Clay. The testing was performed in general accordance with ASTM International (ASTM) designations D4318 (2010b), D854 (2014), and D698 (2012b). The results of the testing are presented in Table 2. Leyden Clay classifies as medium plasticity clay (CL) by the Unified Soil Classification System (USCS).

**Table 2:** Specific gravity, Atterberg limits, and standard proctor values for Leyden Clay.

Leyden Clay (CL)		
Liquid limit	Plastic limit	Plasticity index
40%	20%	20%
Optimum moisture content	17%	
Specific gravity	2.67	
Maximum dry unit weight	111 pcf	

Based on early studies of laboratory test results, the approach of specimen preparation signification affects the strength of disturbed samples. For getting more accurate laboratory test results, saturated samples were prepared by a wet tamping method and performed on isotropically consolidation cyclic triaxial and cyclic hollow cylinder tests.

**Figure 1:**
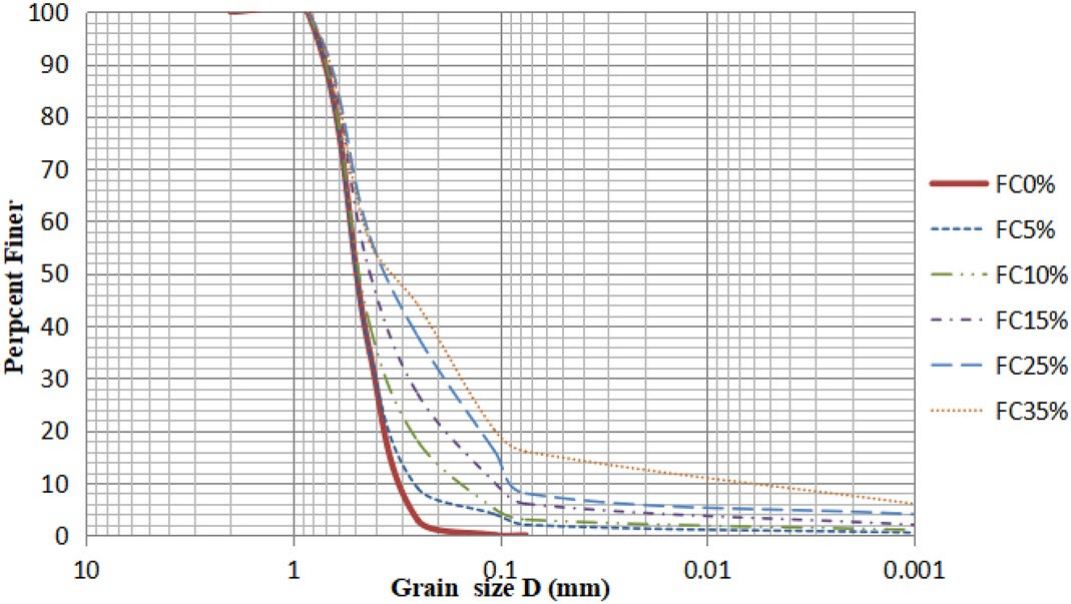
Grain size distribution of soil samples used in this study.

A total of 37 cyclic triaxial and cyclic hollow cylinder tests were run on the uniform medium Monterey sand. A total of 37 cyclic triaxial and cyclic hollow cylinder tests were performed on the mixture of a uniform medium Monterey sand and Leyden clay with five different percentages of fines contents (5%, 10%, 15%, 25%, and 35%) at the same plastic index of 20%. Figure 1 shows the grain size distribution curves for soils with five different percentages of fines content.

To evaluate the correction factor of liquefaction resistance, seven-four samples were prepared at four different relative densities ( 30%, 45%, 50%, and 60%) and consolidated isotropically under the confining water pressure of 15 psi (103 kpa) and 30 psi (207 kpa). All samples were run at five different cyclic stress ratios (0.15, 0.2, 0.25, 0.3, and 0.4) in CTT and CHCT under the same frequency of 0.5 Hz.

### Cyclic triaxial test

A cylindrical soil sample was prepared in a chamber at different relative densities, confining pressure, and back pressure applied, and then loaded axially until failure in the cyclic triaxial test. All soil samples were saturated by applying back pressure. When the Skempton pore pressure parameter, B, was equal to or greater than 0.95, the specimen was considered saturated.

An axial load may be applied to the sample through a rigid top platen. The axial force can be compression or extension: thus the axial stress can be either major or minor principal stress. Usually, the top platen is laid over a porous stone which allows fluid to flow in and out of the specimen. The axial deformation of the specimen is directly monitored by the movement of the piston which is in contact with or connected to the top platen. The lateral deformation is not usually measured. Transducers are used for pore pressure measurement.

In the cyclic triaxial test, the cyclic loading technique can investigate liquefaction resistance. However, some factors, that affect the liquefaction potential of soil samples, are such as specimen preparation method, load waveforms, relative density, and so on. This is in spite of wide recognition of the inability of the test to accurately represent field earthquake stresses^15^.

**Figure 2:**
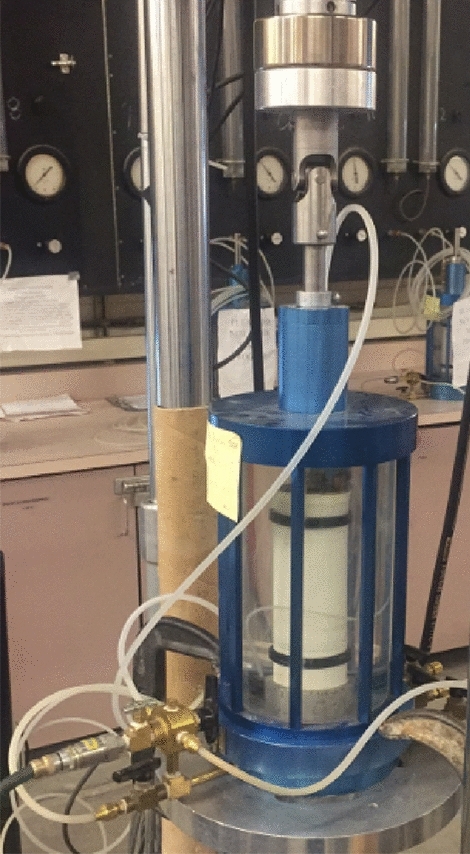
A Typical triaxial cell.

**Figure 3:**
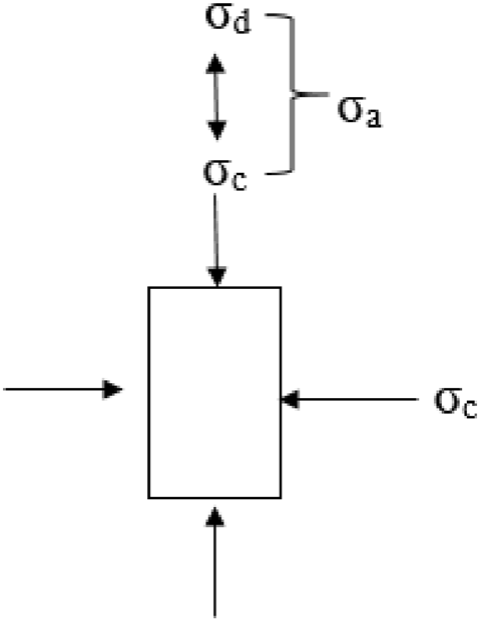
Triaxial stresses.

Based on a chosen stress ratio, cyclic axial stress was applied to the top of the soil sample by using Material Test System series 810. The cyclic axial load was subjected through a ram and top platen, and the cyclic triaxial test apparatus was shown in Fig. 2. During the process of the test, all test results included the cyclic load, strains, and pore water pressure. Figure 3 shows that the soil sample received the stresses in the cyclic triaxial cell. The cyclic deviator (or axial) stress (σ_d_), was applied through the piston. The total axial stress (σ_a_) is equal to deviator stress (σ_d_) plus confining pressure (σ_c_). The lateral stress (σ_c_) is applied through the chamber water. Figure 2 shows the stress application on a triaxial sample (principal stresses, σ_1_, and σ_3_).

### Cyclic hollow cylinder test

**Figure 4:**
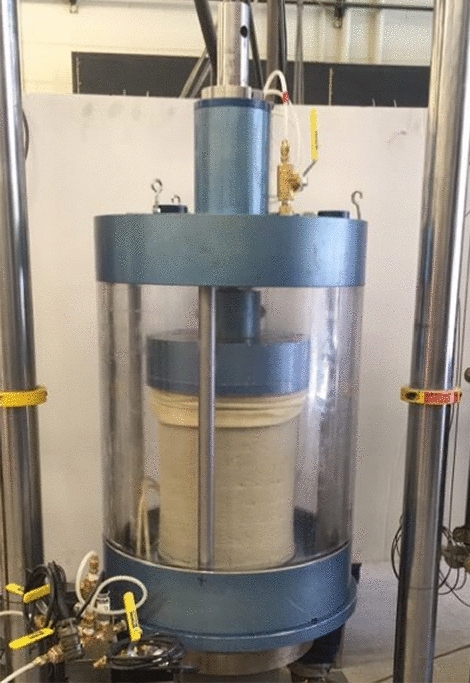
Cyclic torsion hollow cylinder test after liquefaction.

**Figure 5:**
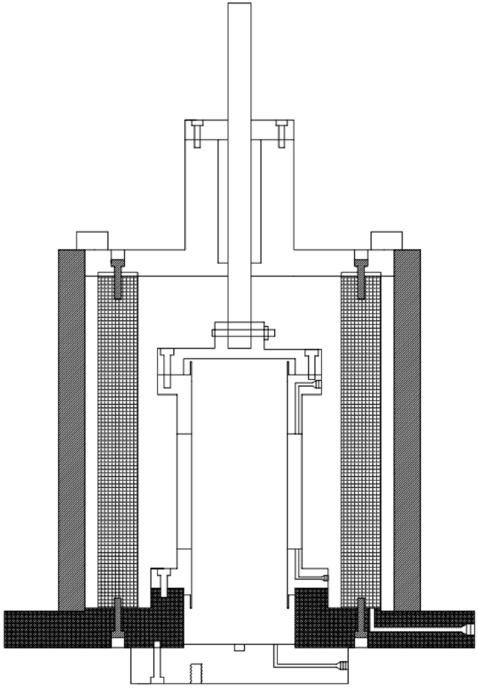
Schematic diagram of the cyclic hollow cylinder test device at the University of Colorado at Denver Geotechnical Laboratory.

The University of Colorado Denver (UCD) Hollow Cylinder Axial/Torsional cell, as shown in Fig. 4. The hollow cylinder cell was designed and manufactured by Chen^16^'s research team at the University of Colorado Denver Geotechnical Laboratory. The schematic diagram of the cyclic hollow cylinder test device was shown in Fig. 5.

A hollow cylindrical soil specimen is enclosed between an inner membrane and an outer membrane. Effective stress can be independently applied to both inner and outer chambers; therefore, inner and outer pressures can be controlled either equally or unequally. The axial load and torque are applied on the top of the specimen and transmitted by a top cap or a pedestal to the specimen.

**Figure 6:**
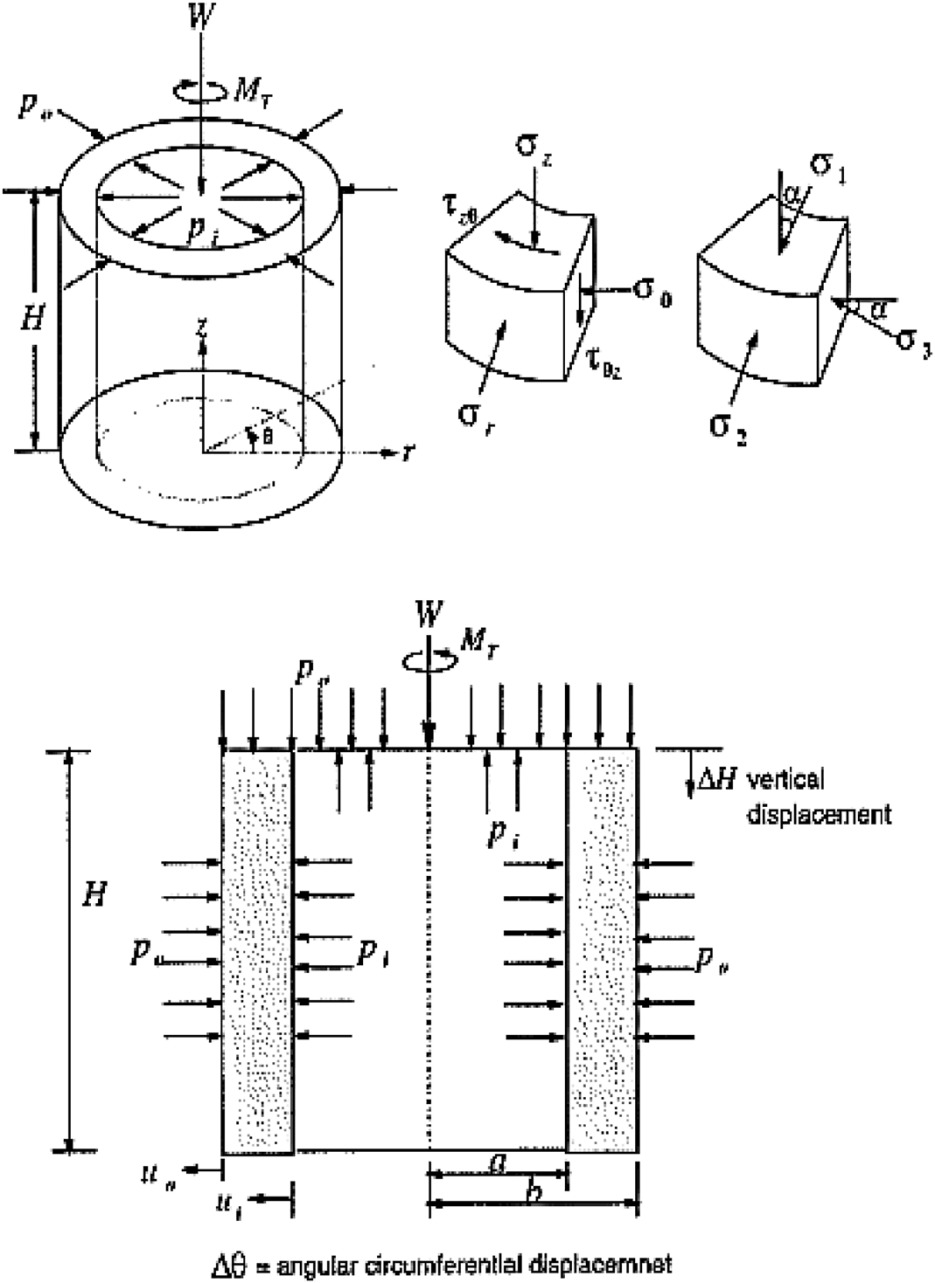
Load and stress conditions for hollow cylinder test (After Hight et al.^8^).

Figure 6 showed that the specimen received stresses in the hollow cylinder test. Axial load was described as W, torque was shown as MT, internal pressure as pi, and external pressure as po. The cyclic torque (MT) is directly applied to the sample through a stiff piston rod and rigid top platen. The average shear stress (τzθ′), the principal stresses, and strains can be calculated from Hight et al.^8^. The axial and rotational displacements and load are monitored via piston movement. The lateral deformation can be monitored but is usually not. Although the different inner and outer confining pressures may be independently applied to the inner and outer chambers, in this study, the same pressure is used and the axial load remains constant throughout the CHC test until the sample liquefies.

### Comparative testing program

A series of isotropically consolidated undrained cyclic triaxial and cyclic hollow cylinder tests were conducted to determine and compare the behavior and liquefaction resistance of soil samples. Thirty-seven cyclic triaxial and thirty-seven hollow cylinder tests were performed.

All seventy-four specimens were prepared at four different relative densities of 30, 45, 50, 60% and tested at frequency of 0.5 Hertz. All triaxial specimens were prepared to attain 2 inches (5.08 cm) in diameter and 4 inches (10.16 cm) in length and hollow cylinder samples to the inner diameter of 8 inches (20.32 cm), the outer diameter of 10 inches (25.4 cm), and height of 10 inches (25.4 cm). Test results are discussed as follows:

### Cyclic torque, cyclic axial load versus number of cycles to liquefaction in CTT and CHCT

**Figure 7:**
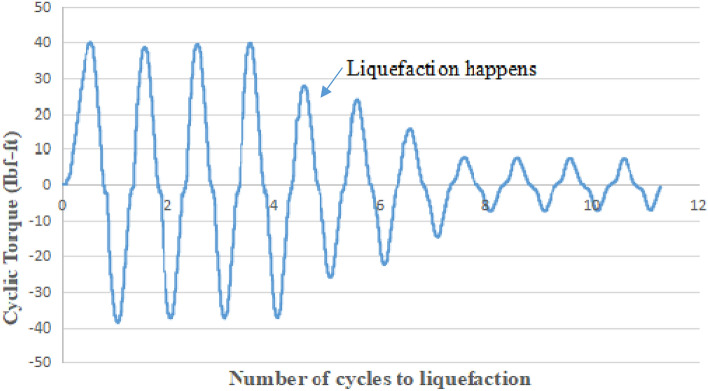
Cyclic torque versus number of cycles to liquefaction in hollow cylinder test.

**Figure 8:**
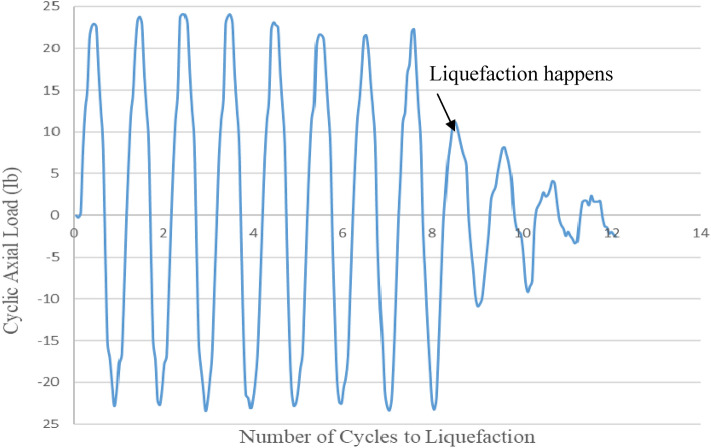
Cyclic axial load versus number of cycles to liquefaction in cyclic triaxial test.

In the CHCT test, a cyclic torsion load of constant amplitude (40Ibf-ft) (54 N.m) was applied with a frequency of 0.5 Hz to a sample of saturated sand. Figure 7 shows cyclic torsion loading versus some cycles to liquefaction in the CHCT test. In the Cyclic Triaxial test, a cyclic axial load of constant amplitude (24 Ib) (107 N) was performed on top of soil specimen with the same frequency which used in CHCT. Figure 8 shows cyclic load versus the number of cycles to liquefaction in the Cyclic Triaxial Test.

In the first 8 cycles of Fig. 8, the amplitude of cyclic loading was held constant without noticeable sample deformation with the increasing number of cycles. It means the soil still was strong. However, in the 5th cycle of Fig. 7, the amplitude of cyclic loading dropped to 25Ibf-ft (34 N m). The amplitude of cyclic loading began to decrease with an increasing number of cycles after the 4th cycle. In a cyclic triaxial test, more numbers cycle to liquefaction than in the CHCT test. In the cyclic triaxial test, the soil specimen was stronger than in the CHCT test under the same stress ratio and relative density.

After the 4th cycle of Fig. 6 and the 9th cycle of Fig. 8, the amplitude of cyclic loading rapidly dropped with the increasing number of cycles. That means both samples were liquefied and too soft in the last few cycles. Compare to both test results in Figs. 7 and 8, the soil sample in the CTT has to apply more number cycles to reach initial liquefaction than in CHCT at the same soil conditions, cyclic stress ratio, and frequency.

### Excess pore water pressure versus number of cycles to liquefaction in CTT and CHCT

**Figure 9:**
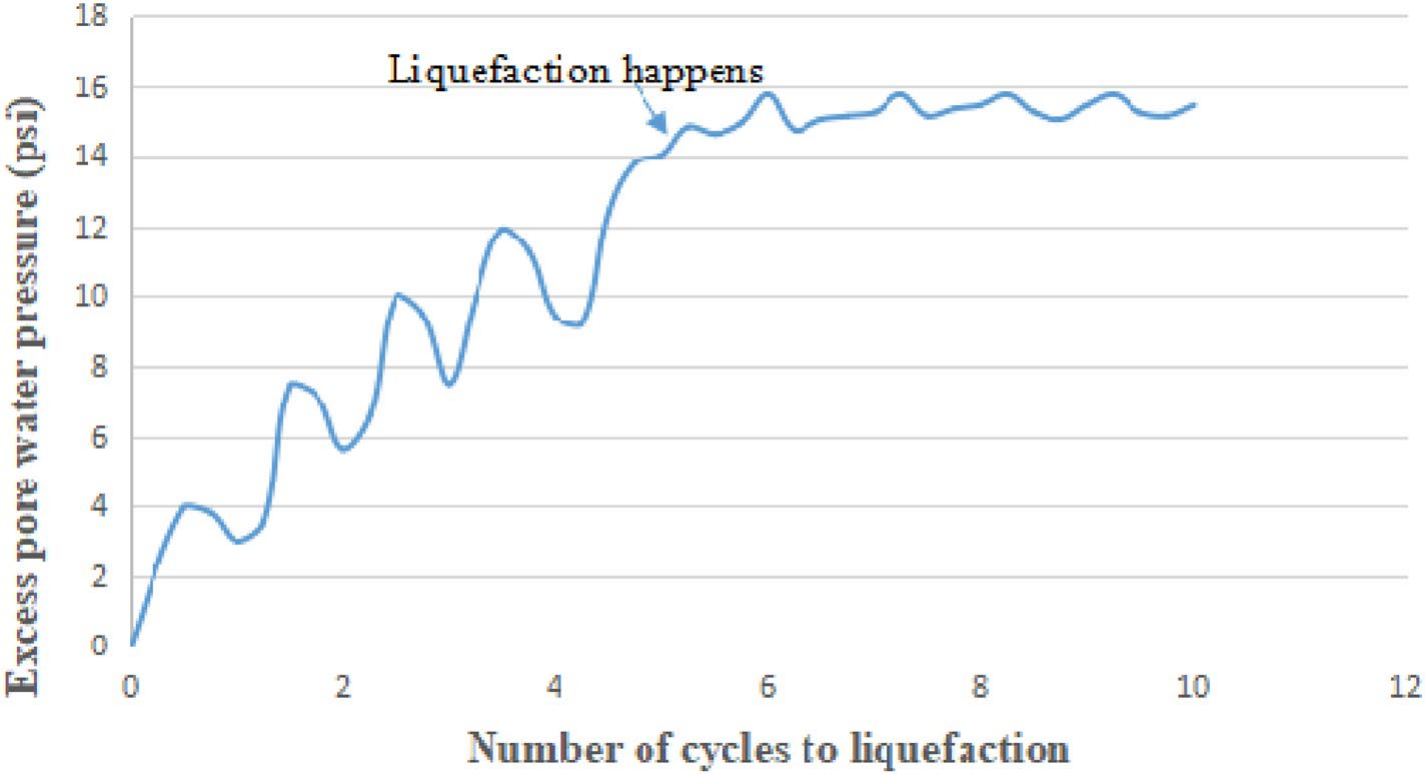
Pore water pressure change versus number of cycles to liquefaction in CHCT.

Figure 9 shows excess pore pressure versus the number of cycles in the Hollow Cylinder test. In the first 4 cycles of cyclic torsion application, the specimen did not show obvious deformation although pore-water pressure built up gradually. However, during the 5th stress cycle, the pore pressure sharply increased to a value equal to the externally applied confining pressure. The soil sample had liquefied, at the same time, the effective confining pressure had been reduced to zero. Over a wide range of strains, the soil could be observed to be in a fluid condition.

**Figure 10:**
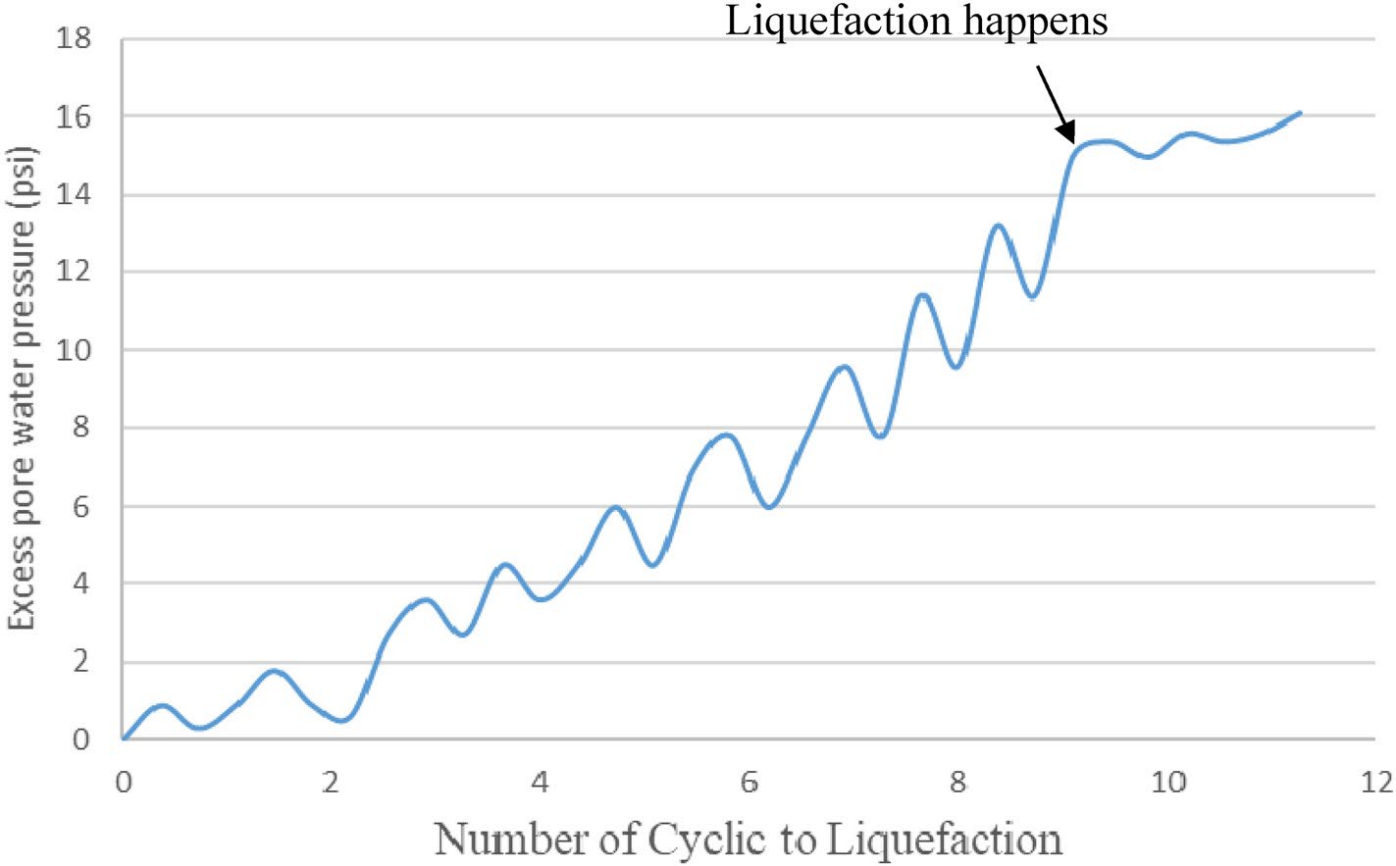
Pore water pressure change versus number of cycles to liquefaction in cyclic triaxial test.

Excess pore-water pressure continues to build up steadily as the number of stress cycles increases until there is a sudden increase denoting the onset of initial liquefaction. The different values of pore-water pressure developed during increases and decreases in deviator stress reflect the influence of the applied stress conditions. Figure 10 shows pore water pressure change versus the Number of cycles to liquefaction in the Cyclic Triaxial Test. Compare to the hollow cylinder test, it needs more stress cycles to make pore pressure increase to a value equal to the externally applied confining pressure. In the CHCT test, the pore water pressure of the soil specimen faster builds up to be equal to effective stress 15 psi (103 kpa) than in the CTT test. When the excess pore pressure reaches 15 psi which is equal to the applied effective stress, the soil liquefaction did not only happen in the 5th cycle of Fig. 9, and also occurs on 9th cycle of Fig. 10.

### Cyclic shear stress vs. shear strain in CHCT and deviator stress vs. axial strain in CTT

**Figure 11:**
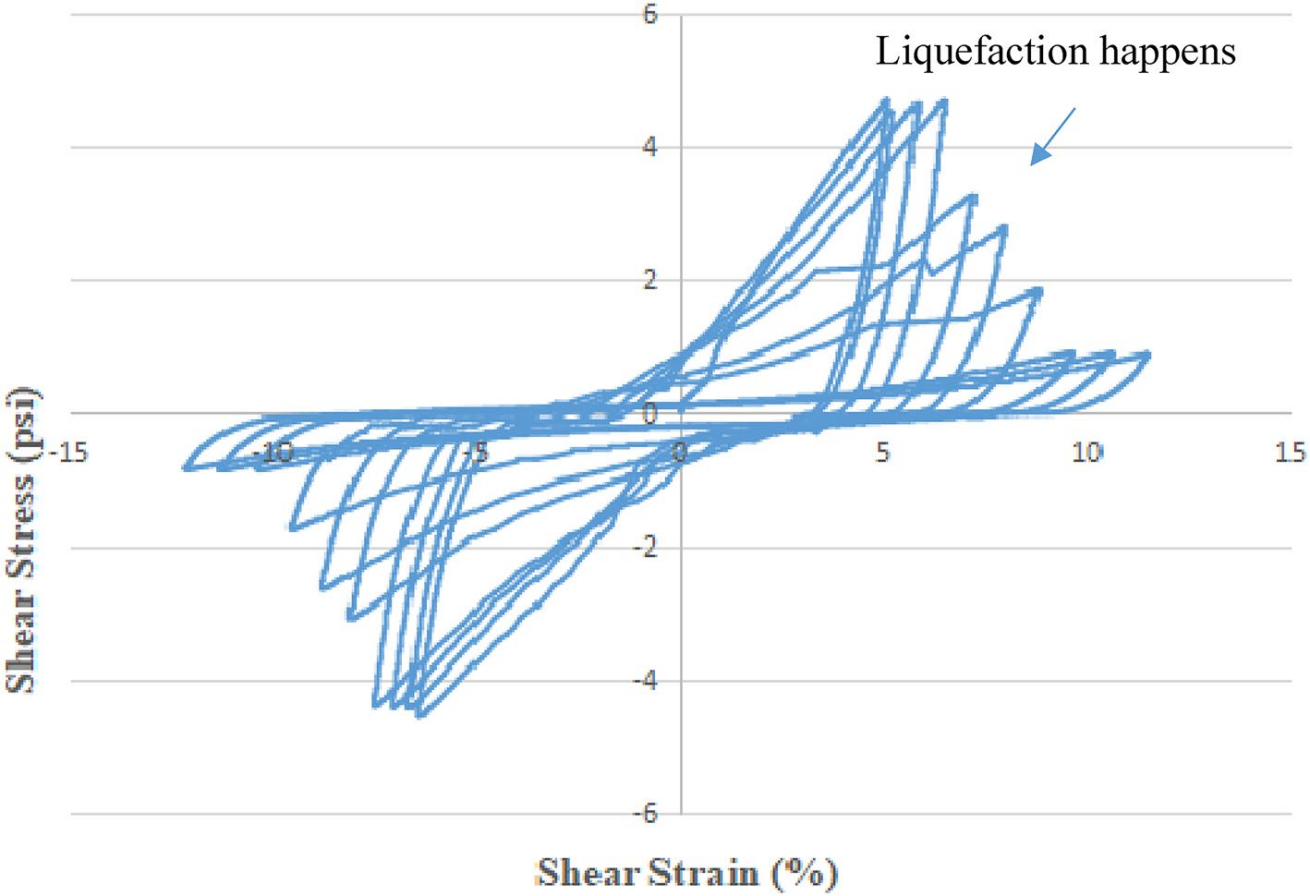
Cyclic shear stress versus shear strain in cyclic hollow cylinder test.

In the first 3 cycles in Fig. 11, the three curves look like one curve, but as the sample approaches failure the strains increase, and the hysteresis loops open up quickly. In the first 3 cycles, the range of shear strain was –5.0% to + 5.0%. It means that the sample was no noticeable deformation. In the last few cycles, the amplitude of deviator stress decreased with increasing the effective mean stress. It can be seen that the sample was softened and large flow deformation took place with the increasing number of cycles. In the 4th cycle, the loops began flat shape, and also the amplitude of deviator stress rapidly dropped by 30%. The sample developed large strains which, in the 5th cycle, exceeded 50% during the last three cycles. That means that the sample had liquefied. In the last two cycles of Fig. 11, the ranges of shear strain were -10% to + 10%.

**Figure 12:**
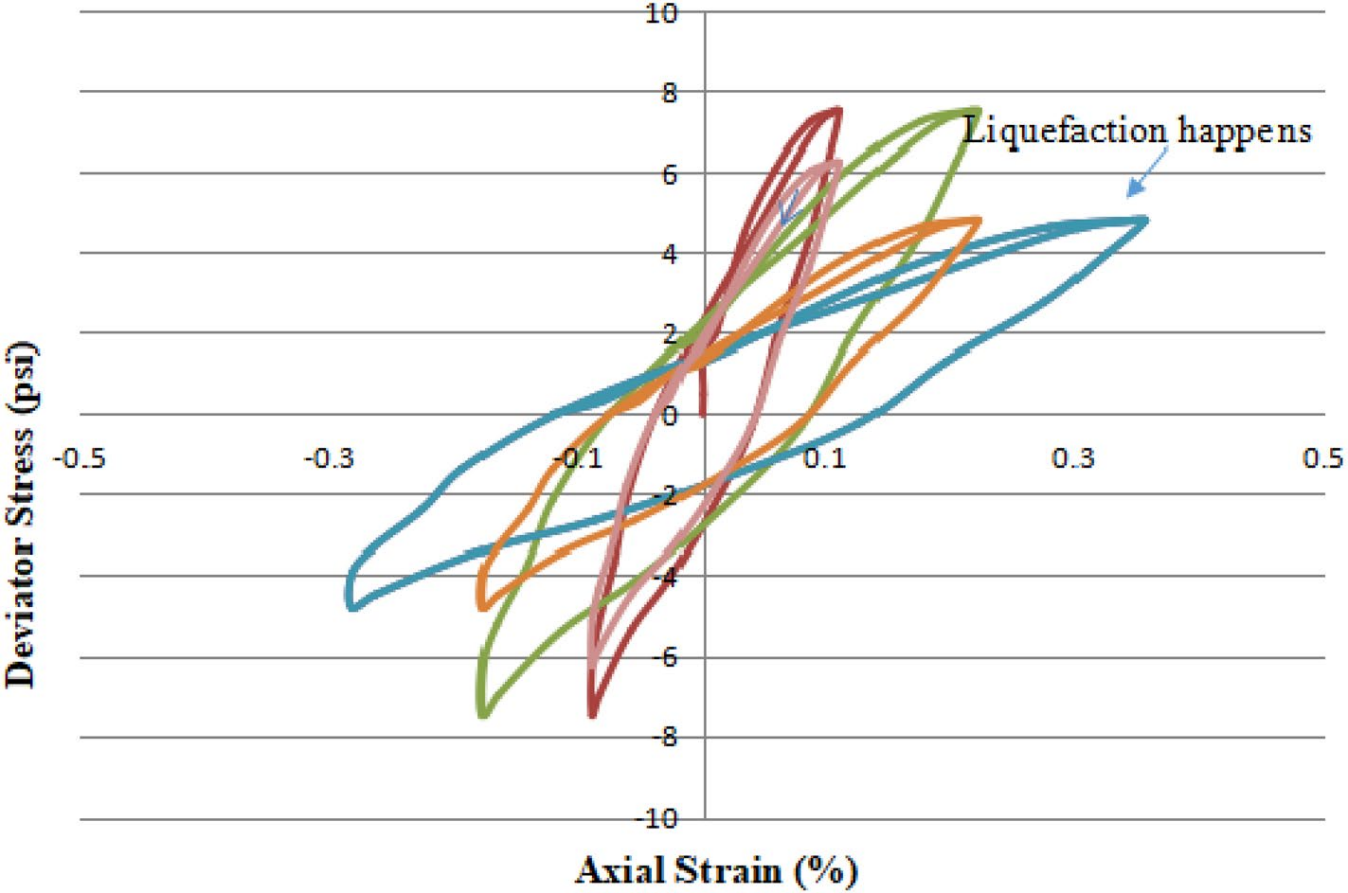
Deviator stress versus axial strain in cyclic triaxial test.

In the CHCT test, cyclic shear stress of constant amplitude (4.5 psi) (31 kpa) was applied with a frequency of 0.5 Hz to a sample of saturated sand. But cyclic deviator stress (7.5 psi) (52 kpa) was applied on the top of the soil specimen with the same frequency of 0.5 Hz. In CTT, the range of axial strain was -0.1% to + 0.1% in the first 3 cycles. It is not noticeable deformation like in the hollow cylinder test. In the last few cycles of Fig. 12, the amplitude of deviator stress decreased with increasing the effective stress, and also soil sample was softened. In the last cycles of Fig. 12, the sample got a larger strain which was -0.3% to 0.3% and the amplitude of deviator stress dropped more than 30%. This shows that the sample had liquefied.

### Stress path in q vs p′ stress space in CTT and CHCT

Thirty-seven isotopically consolidated undrained cyclic triaxial tests and thirty-seven cyclic hollow cylinder tests were performed under stress-controlled loading system on the uniform medium Monterey No. 0/30 sand and it is with four different percentages of fine content. Both tests were conducted at various cyclic stress ratios (CSRs). In this paper, it used the cyclic stress ratio to apply loading on soil samples of the CTT and CHCT tests. Stress path showed the curves of the relationship between cyclic shear stress “q” and mean effective stress “p′”. The value of p′ is equal to one-third of the sum of three mean principal stresses (σ_1_′, σ_2_′, and σ_3_′). The value of q is equal to the subtraction of major principal stress and minor principal stress.

**Figure 13:**
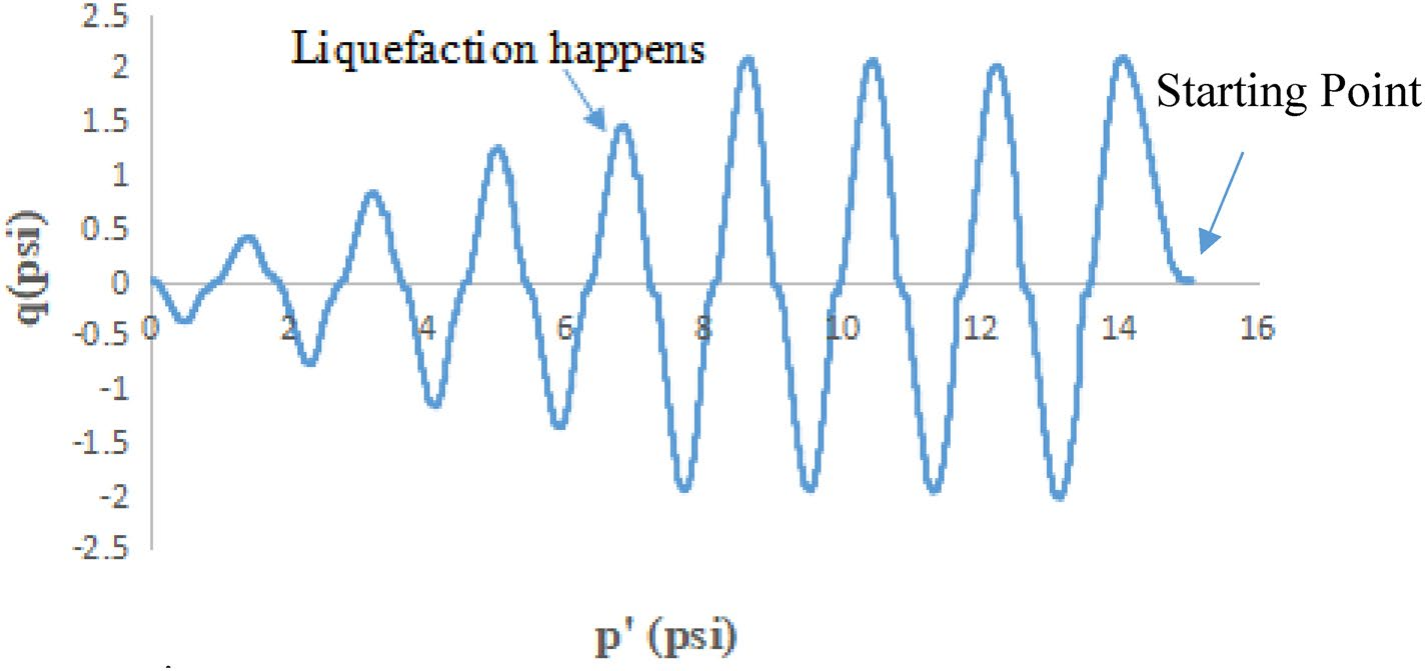
p′ versus q stress path in cyclic hollow cylinder test.

Figure 13 showed the mean effective stress started at 15 psi (103 kpa), and also cyclic shear stress started at 4.5 psi (31 kpa) applied on the sample at the beginning of the Hollow Cylinder test.

In the first 4 cycles of shear stress application, the amplitude of cyclic shear stress kept constant with decreasing the effective mean stress. However, at the beginning of the cyclic triaxial test, the mean effective stress (p′) started at17 psi (117 kpa), and also deviator stress (7.5 psi) (52 kpa) was applied on the sample, and also during the first 8 cycles of stress application, the amplitude of cyclic deviator stress kept constant with decreasing the effective mean stress.

The q-p′ graph reflects the gradual buildup of pore pressure as the effective stress (p′) is reduced until the sample approaches the liquefaction condition at which time the sample starts failing and the amplitude of cyclic shear stress begins to drop. Pore-water pressure equaled the externally applied confining pressure when the soil sample had liquefied. In the 5th cycle of the hollow cylinder test, the amplitude of cyclic shear stress rapidly dropped 33%. However, in the cyclic triaxial test, the amplitude of cyclic deviator stress dropped 10% after the 8th cycle. It means pore-water pressure increased closed to the value of applied confining pressure. After the 5th cycle of the hollow cylinder test (8th cycle of the cyclic triaxial test), the amplitude of cyclic shear stress continued to drop, at the same time, the effective mean stress kept decreasing. It means that the sample turned softer and softer.

**Figure 14:**
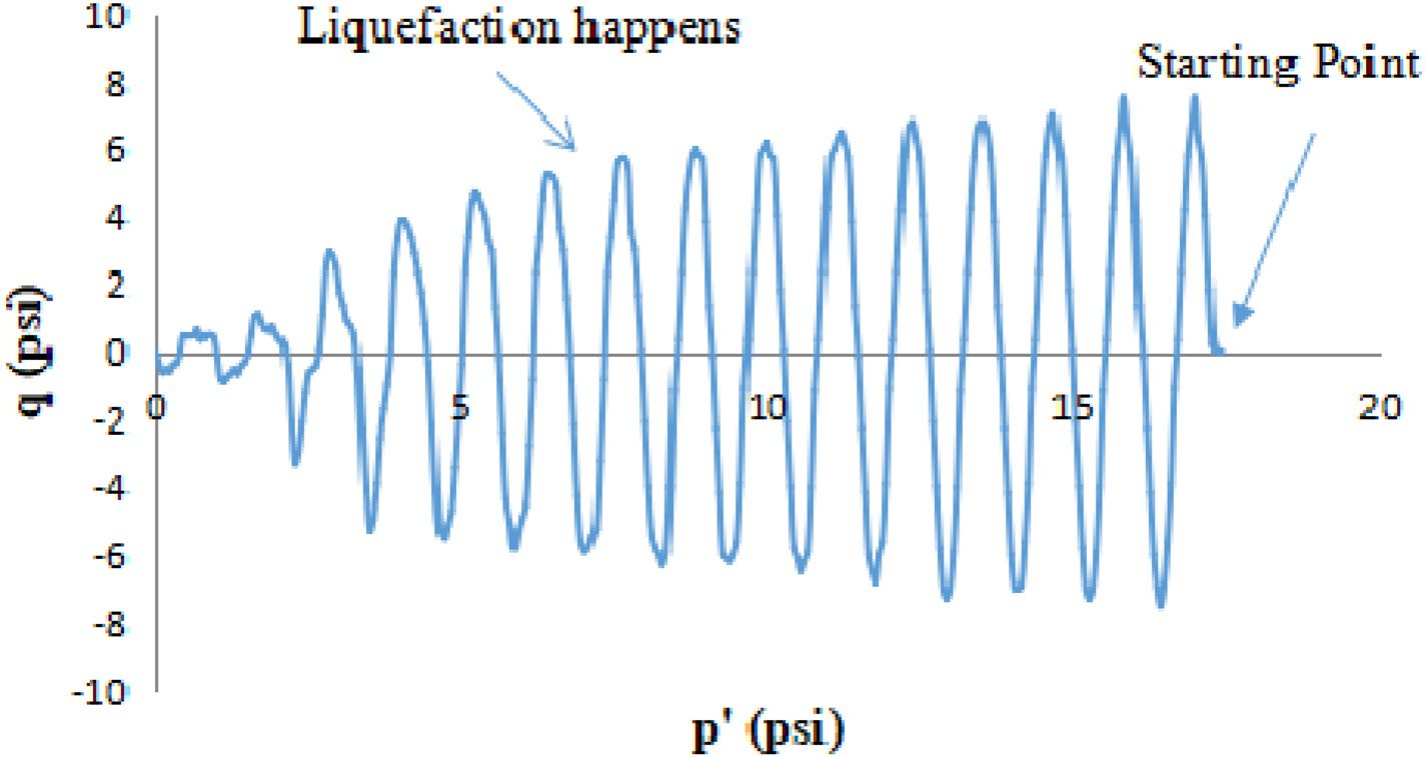
p′ versus q stress path in cyclic triaxial test.

Figure 14 showed that the CTT test started at the mean effective stress (p′) of 17 psi (117 kpa) and deviator stress of 7.5 psi (52 kpa). Through the first 8 cycles of loading, the amplitude of cyclic deviator stress remained constant under the continual decrease of effective mean stress due to continual excess pore pressure increased. The amplitude of cyclic deviator stress dropped about 30% at the 9th cycle of loading. This meant that the sample had liquefied. In Fig. 14, it was shown that it took more cycles of loading to liquefy the same soil in a CTT test than a CHCT under the same stress ratio of 0.25.

### Stress ratio versus number cycles to liquefaction

**Figure 15:**
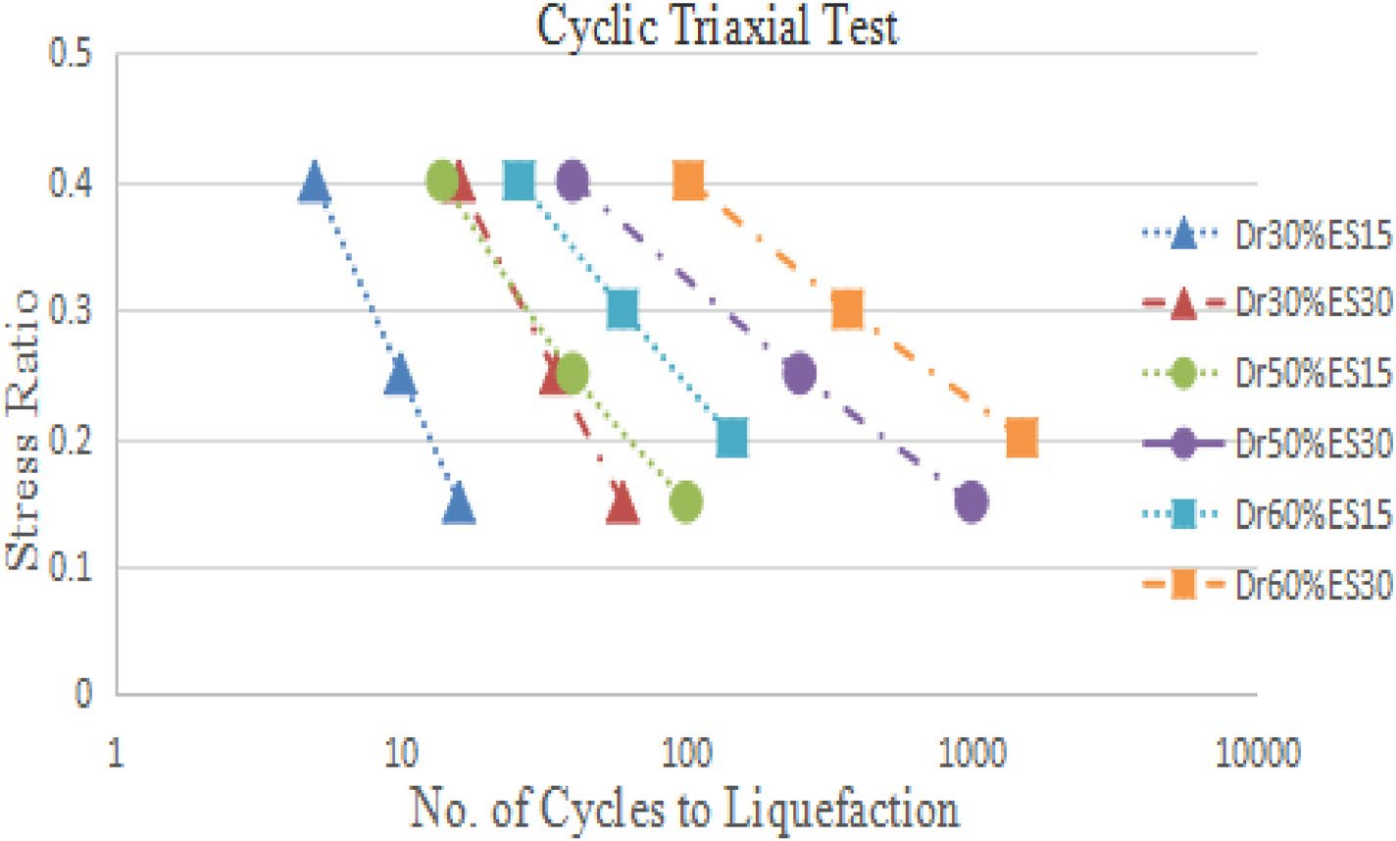
Stress ratio vs number of cycles to liquefaction in CTT.

**Figure 16:**
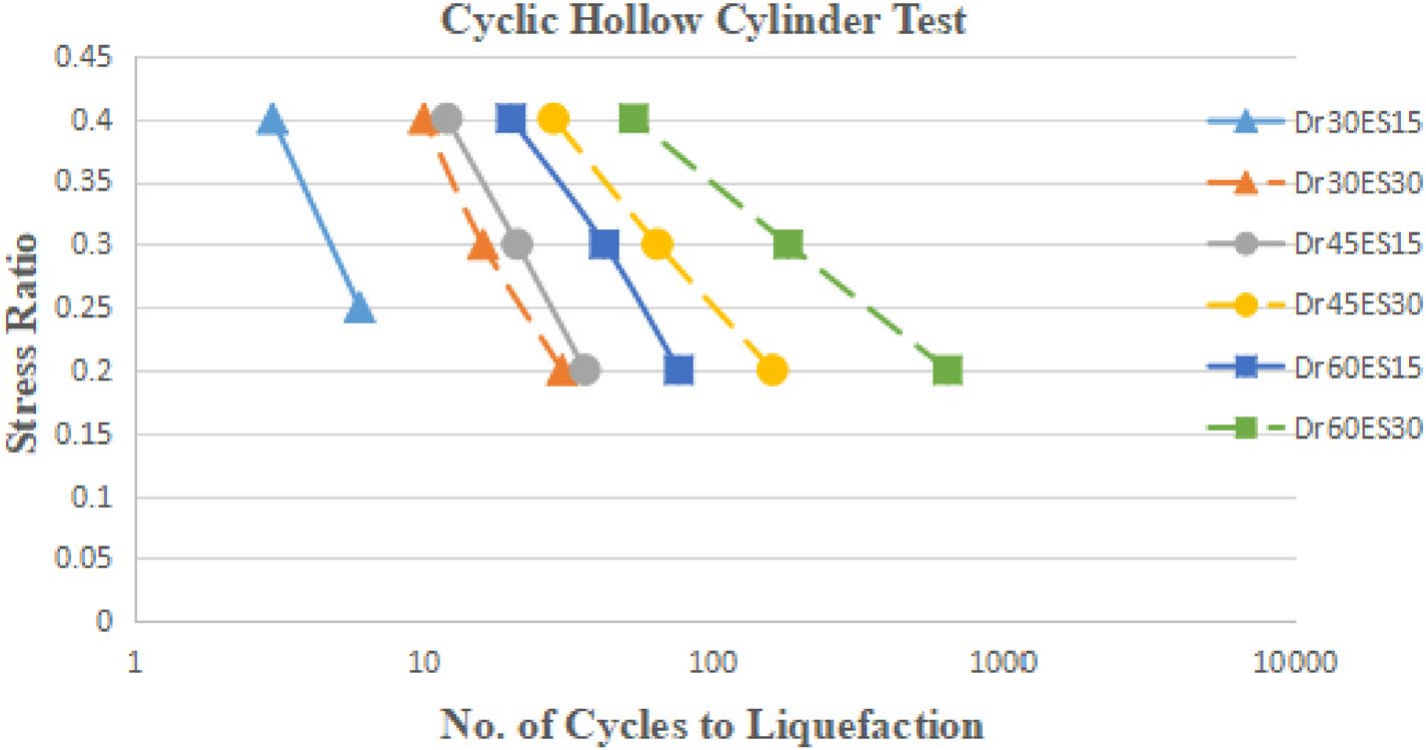
Stress ratio vs number of cycles to liquefaction in CHCT.

17 CHCT tests and 18 CTT tests results have performed the curves of stress ratio vs the number of cyclic to liquefaction. In Fig. 15, eighteen CTT test results showed that density increased with the increasing number of cycles to liquefaction. The bigger the effective stress (E.S.), the more cycles to liquefaction. It showed that the cyclic stress ratio decreased linearly with the increasing number of cycles to liquefaction in Fig. 15. In Fig. 16, seventeen CHCT test results showed the same conclusions from the CTT test results.

**Figure 17:**
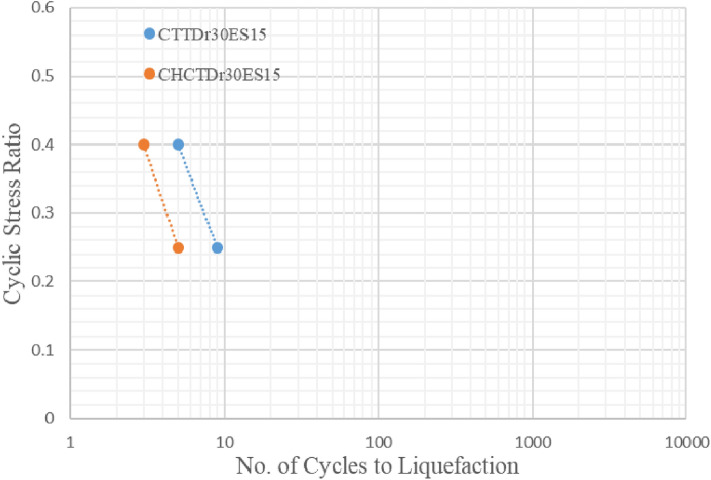
stress ratio vs number of cycles to liquefaction for samples (Dr 30%FC0ES15) in CTT and CHCT.

**Figure 18:**
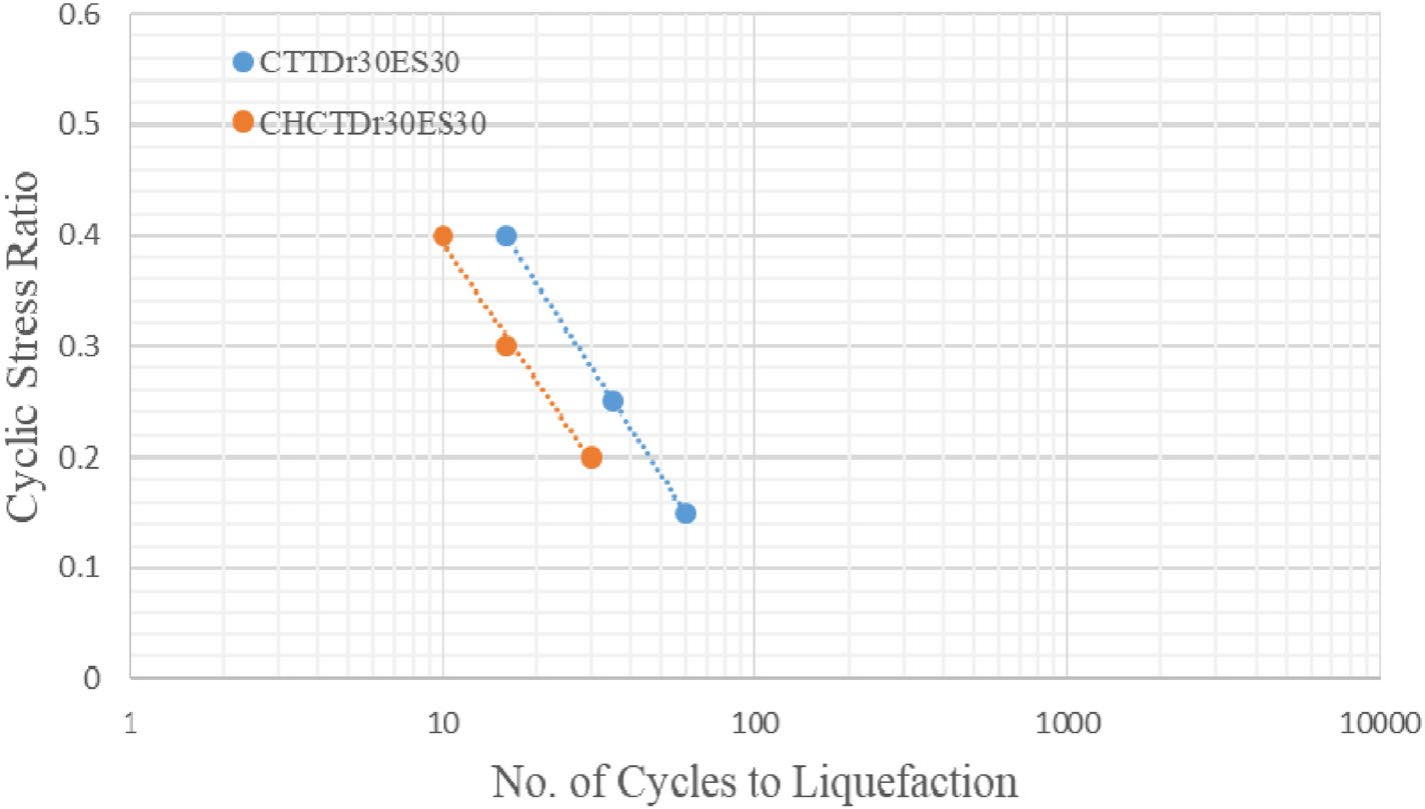
Stress ratio vs number of cycles to liquefaction for samples (Dr 30% FC0ES30) in CTT and CHCT.

**Figure 19:**
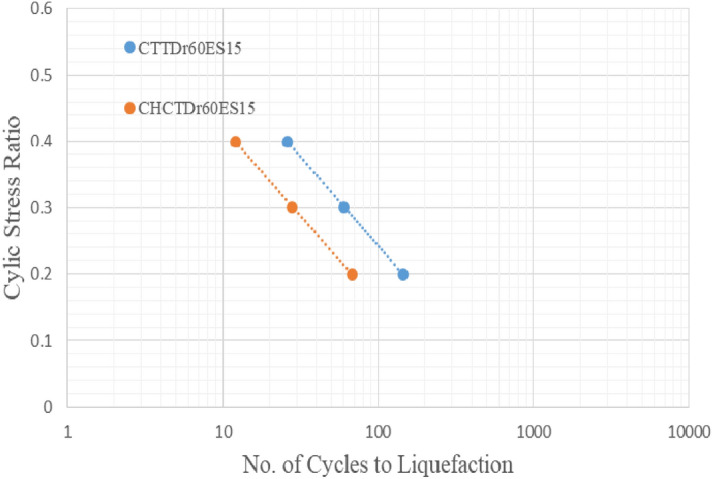
Stress ratio vs number of cycles to liquefaction for samples (Dr 60% FC0ES15) in CTT and CHCT.

**Figure 20:**
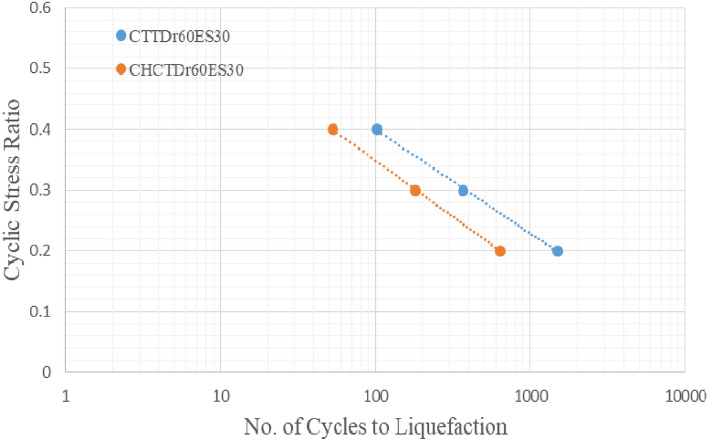
Stress ratio vs number of cycles to liquefaction for samples (Dr 60% FC0ES30) in CTT and CHCT.

Figures 17, 18, 19 and 20 showed that comparing two types of test results on the same sample and effective stress. The curves indicated samples in CHCT had fewer cycles to liquefaction than in CTT under the same cyclic stress ratio in Figs. 17, 18, 19 and 20.

### Correction factors

Seed^17^ found that the cyclic stress ratio causing liquefaction under multidimensional shaking conditions in the field is related to the cyclic stress ratio causing liquefaction of a triaxial test sample in the laboratory. Table 3 shows that many researchers evaluated the values of C_r_ under different conditions. The values of C_r_ formulated the following expression:

$$\left( {\frac{{\tau_{h} }}{{\sigma_{v}^{^{\prime}} }}} \right){\text{l-field}} \approx {\text{C}}_{{\text{r}}} \left( {\frac{{\sigma_{dc} }}{{2\sigma_{a} }}} \right){\text{l-triaxial}}$$

**Table 3:** Values of correction factor C_r_ by earlier researchers.

Researchers	Equations	C_r_ for K_0_ = 0.4	C_r_ for K_0_ = 1.0
Finn et al.^18^	C_r_ = (1 + K_0_)/2	0.7	1.0
Seed and Peacock^19^	Varies	0.55–0.72	1.0
Castro^20^	C_r_ = 2(1 + 2K_0_)/3	0.69	1.15

In this study a series of the cyclic hollow cylinder and cyclic triaxial tests were performed to establish the following relation between the cyclic liquefaction resistance stresses ratios of these two types of tests, the former closely simulates the field shear wave propagation during earthquake shaking. CHCT not only has much more uniform distributions of stress and strain but also can be more ideally simulated field conditions in the laboratory test. The main objective is to evaluate the correction factor, Cr′, in the following equation:

$$\left( {\frac{{\tau_{h} }}{{\sigma_{v}^{^{\prime}} }}} \right) {\text{l-hollow cylinder}} \approx {\text{C}}_{{\text{r}}}^{^{\prime}} \left( {\frac{{\sigma_{dc} }}{{2\sigma_{a} }}} \right){\text{l-triaxial}}$$

The results of this comparison are summarized in Table 4. As may be seen, the Cr′ values range from 0.46 to 0.63 at the same relative densities of 30% and 60%, which falls within the range of values that Seed assessed in Table 3. The average value of Cr′ is 0.508 based on both test results. For the samples with different fines content, Table 5 showed the results of the comparison. The Cr′ values range from 0.32 to 0.93 in soil samples with different fines content. For 30% of relative density in Table 5, The Cr′ values range from 0.5 to 0.93, but the Cr′ values at 60% of relative density range from 0.32 to 0.44.

**Table 4:** Cr′ values on medium clean produced in laboratory by CTT and CHCT.

Number of cycles to liquefaction		Effective stress (psi)		Stress ratio		Relative density (%)	Cr′
CTT	CHCT	CTT	CHCT	CTT	CHCT		
9	5	15	15	0.25	0.25	30	0.56
5	3	15	15	0.4	0.4	30	0.60
60	30	30	30	0.15	0.2	30	0.50
35	16	30	30	0.25	0.3	30	0.46
16	10	30	30	0.4	0.4	30	0.63
100	28	15	15	0.2	0.15	50 (45)	0.28
40	15	15	15	0.3	0.25	50 (45)	0.38
14	8	15	15	0.4	0.4	50 (45)	0.57
1000	160	30	30	0.2	0.2	50 (45)	0.16
250	64	30	30	0.3	0.3	50 (45)	0.26
40	28	30	30	0.4	0.4	50 (45)	0.70
145	68	15	15	0.2	0.2	60	0.47
60	28	15	15	0.3	0.3	60	0.47
26	12	15	15	0.4	0.4	60	0.46
1500	643	30	30	0.2	0.2	60	0.43
368	181	30	30	0.3	0.3	60	0.49
102	53	30	30	0.4	0.4	60	0.52

**Table 5:** C_r_′ values on the mixture samples produced in laboratory by CTT and CHCT.

Number of cycles to liquefaction		Effective stress (psi)		Cyclic stress ratio		Fine content (%)		Target relative density (%)	C_r_′
CTT	CHCT	CTT	CHCT	CTT	CHCT	CTT	CHCT		
10	8	15	15	0.4	0.4	5	5	30	0.8
30	28	30	30	0.4	0.4	5	5	30	0.93
10	7	15	15	0.3	0.3	10	10	30	0.7
30	26	30	30	0.3	0.3	10	10	30	0.87
10	7	15	15	0.2	0.2	15	15	30	0.7
35	26	30	30	0.2	0.2	15	15	30	0.74
14	7	15	15	0.3	0.3	25	25	30	0.5
36	26	30	30	0.3	0.3	25	25	30	0.72
14	8	15	15	0.4	0.4	35	35	30	0.57
32	27	30	30	0.3	0.3	35	35	30	0.84
55	19	15	15	0.4	0.4	5	5	60	0.35
90	40	30	30	0.4	0.4	5	5	60	0.44
56	18	15	15	0.3	0.3	10	10	60	0.32
95	39	30	30	0.3	0.3	10	10	60	0.41
55	18	15	15	0.2	0.2	15	15	60	0.33
98	38	30	30	0.2	0.2	15	15	60	0.39
60	19	15	15	0.3	0.3	25	25	60	0.32
97	38	30	30	0.3	0.3	25	25	60	0.39
48	18	15	15	0.4	0.4	35	35	60	0.38
88	39	30	30	0.4	0.4	35	35	60	0.44

### Statistical regression models for Cr′

A statistical model was developed to portend the correction factor of soil liquefaction resistance between CTT and CHCT tests by using all laboratory test results.

A total of thirty-four test results data on clean soil samples was applied to produce the independent variables. In the statistical model, the correction factor Cr′ was chosen as the dependent variable. Through the variable reduction analysis, the independent variables were reduced to the final three: relative density in cyclic triaxial test, Dr_(CTT)_, number of cycles to liquefaction in cyclic triaxial test, No. _(CTT)_, and relative density in cyclic hollow cylinder test, Dr_(CHCT)_.

By using the excel program, linear regression analysis was performed with one dependent variable Cr′, and three independent variables. The R^2^- value for the regression equation is 0.84. A linear regression model involving Dr_(CTT)_, No. _(CTT)_, and Dr_(CHCT)_ as predictors were obtained as follows:

$${\text{C}}_{{\text{r}}}^{^{\prime}} = {1}.{136} + 0.0{15}*{\text{D}}_{{{\text{r}}({\text{CTT}})}} - 0.00{2}*{\text{No}}._{{({\text{CTT}})}} - 0.0{3}0*{\text{D}}_{{{\text{r}}({\text{CHCT}})}}.$$

Based on the cyclic triaxial and cyclic hollow cylinder test results on all thirty-four clean soil samples, one statistical model was performed, involving three significant independent variables and one dependent variable. In the statistical model, D_r(CTT)_, No. _(CTT)_, and D_r(CHCT)_ can the forecast correction factor of soil liquefaction resistance between CTT and CHCT tests.

Forty test data on soil samples with different fines content were involved to obtain the independent variables. In the statistical model, the correction factor C_r_′ was chosen as the dependent variable. Through the variable reduction analysis, the independent variables were reduced to the final four: fine content (%) in cyclic triaxial test and cyclic hollow cylinder test, FC_(CTT&CHCT),_ relative density in cyclic triaxial test and cyclic hollow cylinder test, D_r(CTT&CHCT)_, number of cycles to liquefaction in cyclic triaxial test, No. _(CTT)_ and a number of cycles to liquefaction in cyclic hollow cylinder test, No. _(CHCT)_.

By using the excel program, linear regression analysis was performed with one dependent variable C_r_′, and three independent variables. The R^2^-value for the regression equation is 0.95. A linear regression model involving FC_(CTT&CHCT)_, D_r(CTT&CHCT)_, No._(CTT)_, and No. _(CHCT)_ as predictors were obtained as follows:

$${\text{C}}_{{\text{r}}}^{^{\prime}} = 0.{775} - 0.00{2}*{\text{FC}}_{{({\text{CTT}}\& {\text{CHCT}})}} - 0.00{4}*{\text{D}}_{{{\text{r}}\left( {{\text{CTT}}\& {\text{CHCT}}} \right) }} - 0.00{8}*{\text{No}}._{{({\text{CTT}})}} + 0.0{19}*{\text{No}}._{{({\text{CHCT}})}}$$

Based on the cyclic triaxial and cyclic hollow cylinder test results on all forty soil samples with different fines content, one statistical model was performed, involving four significant independent variables and one dependent variable. In the statistical model, FC_(CTT&CHCT)_, Dr_(CTT&CHCT)_, No._(CTT)_, and No. _(CHCT)_ can the forecast correction factor of soil liquefaction resistance between CTT and CHCT tests.

## Discussion

Cyclic isotopically consolidated undrained cyclic triaxial and hollow cylinder tests were performed to evaluate the correction factor of liquefaction resistance. Although there are two types of soil specimens involved in this paper, Monterey No. 0/30 sand is a uniform clean, and common research soil sample.

For evaluating liquefaction resistance, the cyclic hollow cylinder test is an ideal laboratory testing apparatus that can closely simulate the field’s initial stress and loading conditions.

With appropriate dimensions, the distribution of stresses and strains in the CHCT sample is much more uniform than that of the cyclic triaxial test and CHCT is superior to CTT in the evaluation of liquefaction resistance of soil, but CTT is much more prevalent due to its simplicity. CHCT can apply perfectly uniform conditions of stress and strain, and also can more ideally simulate field situations. Thus, CHCT is considered an ideal test for the evaluation of liquefaction resistance. Liquefaction resistance of soil samples in CTT can use the correction factor Cr′ to assess the liquefaction potential of geo-structures and superstructures built on liquefiable soils.

Differences often exist between laboratory reconstituted samples and specimens in the field. However, the correction factor Cr′ seems like a bridge that can be used to convert liquefaction resistance between laboratory and field tests. It can help engineers better understand the field evaluation of liquefaction resistance of soils based on the laboratory test results. The correction factor gave engineers more accurate testing results for the field assessment of the liquefaction potential of soil.

The geotechnical engineer always uses the laboratory testing programs to be compared with real-world information and determines the laboratory test results “how good it is”. So the correction factor Cr′ seems like a bridge that can be used to convert liquefaction resistance between laboratory and field tests. It can help the geotechnical engineers have more confidence to determine the evaluation of liquefaction resistance of a soil sample from the field based on the statistical regression models and laboratory test results. The correction factor gave engineers more accurate testing results for the field assessment of the liquefaction potential of soil.

CHC can be used to calibrate the CTT liquefaction resistance and evaluate the reduction factor, Cr^21^ to convert CTT liquefaction resistance to the cyclic simple shear condition. In this sense, CTT is used in production and CHC is for quality control and both tests have their edges in the liquefaction resistance evaluation of soils. When using CTT to assess the liquefaction resistance of soil, the correction factor Cr′ can be used to evaluate the field liquefaction resistance for seismic safety assessment of geo-structures and superstructures supported on liquefiable soils.

## Conclusions

In this study, seventy-four cyclic triaxial and cyclic hollow cylinder tests were performed to investigate the correction factor of liquefaction resistance.

Two different types of soil samples were involved. One soil sample used in the present experimental investigation was a uniform medium Monterey No. 0/30 clean sand. Another soil sample is the mixture of a uniform medium Monterey No. 0/30 sand and Leyden Clay (M-L) from Golden Colorado, with five different percentages of fines content and the same plastic index of 20%.

In this paper, some conclusions are summarized as follows:

1. Cyclic hollow cylinder tests and cyclic triaxial tests are good laboratory test devices to evaluate soil liquefaction resistance. CHCT has a better ability to evaluate the field liquefaction resistance of soil than the cyclic triaxial test because CHCT can simulate the simple shear condition during seismic loading, and also sample in CHCT has much more uniform stresses and strains.
2. CHCT can serve as calibrator to CTT tests for simulating earthquake-loading and evaluating the correlation factor, “C_r_”,
3. Cyclic stress ratio dropped linearly with an increasing number of cycles to liquefaction in both test results. The density soil sample and effective stress had a signification effect on soil liquefaction resistance.
4. The correction factor evaluated ranges from 0.46 to 0.63 under the same relative densities of 30% and 60% of Monterey No. 0/30 sand. For Monterey No. 0/30 sand with different fines content, the C_r_′ values range from 0.5 to 0.93 at 30% of relative density, and C_r_′ values at 60% of relative density range from 0.32 to 0.44.
5. The correction factor of clean soil samples’ liquefaction resistance can be predicted by the regression model: C_r_′ = 1.136 + 0.015*D_r(CTT)_ − 0.002*No. _(CTT)_ − 0.030*D_r(CHCT)_ which involves C_r_′, as indicator of the correction factor of soil liquefaction resistance while D_r(CTT)_, No. _(CTT)_, and D_r(CHCT)_ as indicators of relative density in cyclic triaxial test, number of cycles to liquefaction in cyclic triaxial test, and relative density in cyclic hollow cylinder test.
6. The correction factor of soil samples with fines content liquefaction resistance can be predicted by the regression model: C_r_′ = 0.775 − 0.002*FC_(CTT&CHCT)_ − 0.004*D_r(CTT&CHCT)_ − 0.008*No. _(CTT)_ + 0.019*No._(CHCT)_ which involves C_r_′, as indicator of the correction factor of soil liquefaction resistance while FC_(CTT&CHCT),_ D_r(CTT&CHCT),_ No. _(CTT)_ and No. _(CHCT)_ as indicators of fine content (%) in cyclic triaxial test and cyclic hollow cylinder test, relative density in cyclic triaxial test and cyclic hollow cylinder test, number of cycles to liquefaction in cyclic triaxial test and number of cycles to liquefaction in cyclic hollow cylinder test.

## Data Availability

All data, models, and code generated or used during the study appear in the submitted article.
